# Torsion d'annexe en cours de grossesse: à propos d'un cas à l'Hôpital Central de Yaoundé, Cameroun

**DOI:** 10.11604/pamj.2014.17.39.3006

**Published:** 2014-01-20

**Authors:** Jeanne Hortence Fouedjio, Jovanny Tsuala Fouogue, Florent Ymele Fouelifack, Charlette Nangue, Zacharie Sando, Robinson Enow Mbu

**Affiliations:** 1 Unité de Gynécologie-Obstétrique de l'Hôpital Central de Yaoundé, Yaoundé, Cameroun; 2 Département de Gynécologie-Obstétrique de la Faculté de Médecine et des Sciences Biomédicales de l'Université de Yaoundé 1, Yaoundé, Cameroun; 3 Laboratoire d'Anatomie Pathologique de l'Hôpital Central de Yaoundé, Yaoundé, Cameroun; 4 Département de Sciences Morphologiques et d'Anatomie Pathologique de la Faculté de Médecine et des Sciences Biomédicales de l'Université de Yaoundé 1, Yaoundé, Cameroun

**Keywords:** Kyste ovarien, grossesse, torsion, annexe, avortement, ovarian cyst, pregnancy, torsion, appendages, abortion

## Abstract

Les kystes ovariens sont dans la majorité des cas asymptomatiques et peuvent être de découverte fortuite lors d'une échographie. Ils ne deviennent symptomatiques que lorsque survient une complication. Nous présentons un cas de torsion d'annexe gauche diagnostiqué à 8 semaines et 4 jours de grossesse. Nous avons réalisé une annexectomie Suivie de l'administration de progestérone retard à la dose 500 mg par jour. L'évolution a été marquée par la survenue d'un avortement au cinquième jour post opératoire. L'analyse anatomopathologique de la masse chirurgicale a conclu à une apoplexie ovarienne. L'ablation chirurgicale du corps jaune au premier trimestre de la grossesse pose le problème du maintien de celle - ci et devrait être présente à l'esprit des praticiens avant toute chirurgie pelvienne pendant cette période.

## Introduction

Les masses annexielles sont de plus en plus fréquentes avec la réalisation et la qualité sans cesse croissantes de l'échographie en grossesse [1]. Le traitement conservateur se discute en dehors de l'urgence; cependant la survenue des complications aiguës telles que la torsion d'annexe (TA), impose la réalisation d'actes chirurgicaux plus ou moins invasifs [2]. La torsion d'annexe en cours de grossesse est une entité rare survenant majoritairement aux cours des deux premiers trimestres de la grossesse (70 à 90%) [3, 4]. Elle est favorisée par l'hyperstimulation ovarienne observée dans la procréation médicalement assistée [5]. Certains auteurs plaident en faveur de la préservation de l'ovaire malgré son apparence nécrotique car sa fonction serait préservée dans 88 à 100% des cas [6] ; malgré la grande capacité de récupération de l'ovaire, une chirurgie conservatrice ne se conçoit que dans les cas vus et traités précocement ce qui est très rarement le cas dans les contextes pauvres en ressources [7]. Nous présentons le cas d'une torsion d'annexe au premier trimestre de grossesse.

## Patiente et observation

Mademoiselle N.A.V. âgée de 24 ans, gravida 2 para 1001, à huit semaines et quatre jours de grossesse, a été reçue aux urgences pour une douleur de la fosse iliaque droite évoluant depuis 12 heures de temps.

L'histoire révèle la survenue brutale d'une douleur de la fosse iliaque droite ayant réveillé la patiente; cette douleur était continue, d'intensité élevée, à type de torsion permanente, à irradiation hypogastrique, sans position antalgique et associée à des vomissements bilieux. Après un traitement à base d'antispasmodiques sans amélioration dans un dispensaire, la patiente à été référée vers notre service d'urgence.

Notre patiente a eu ses ménarches à 13 ans; son cycle menstruel a une durée de 24 jours et ses règles durent quatre à six jours. Depuis son premier rapport sexuel à l'âge de 19 ans, elle a eu cinq partenaires sexuels. Sa méthode contraceptive est le préservatif masculin. Elle n'a jamais eu d'infection sexuellement transmissible. Sa première grossesse s'est achevée par l'accouchement normal à terme d'un nouveau-né de sexe féminin deux ans plus tôt. Au moment de l'admission elle portait une grossesse de huit semaines et quatre jours.

Notre patiente ne souffrait d'aucune pathologie médicale chronique et ne consommait ni alcool ni tabac ; elle ne présentait aucune allergie. Elle n'avait jamais subi d'intervention chirurgicale. Elle est du groupe sanguin A rhésus positif. Au moment de la survenue de la douleur ayant motivé la consultation, aucun traitement médicamenteux n'était en cours. Les géniteurs de notre patiente étaient en vie et en bonne santé apparente au moment de l'anamnèse. Elle est troisième au sein d'une fratrie de cinq, les autres étant tous bien portant. Son partenaire âgé de 34 ans et sa fille de deux ans étaient en bonne santé apparente.

En plus du motif de consultation l'enquête des systèmes n'a retrouvé que des céphalées en casque d'intensité modérée.

A l'examen physique, la patiente était consciente, bien orientée dans le temps et l'espace avec un état général conservé mais au faciès algique. Les paramètres vitaux étaient les suivants: pression artérielle : 115/80 millimètres de mercure ; température de 37,4 degrés Celsius, pouls de 88 pulsations par minutes ; fréquence respiratoire de 22 cycles par minute, et un poids de 59 kilogrammes. Les conjonctives étaient bien colorées et les sclérotiques étaient anictériques. La cavité buccale était rose propre et humide. L'examen des seins était normal et nous avons retrouvé une polypnée superficielle sans râles ainsi qu'une tachycardie sans souffle. L'abdomen était mobile avec la respiration et présentait une voussure de la fosse iliaque droite. La palpation superficielle n'a pas retrouvé d'hyperesthésie cutanée. Nous avons retrouvé une masse ovalaire douloureuse de la fosse iliaque gauche, de surface lisse de contours difficiles à apprécier à cause de la douleur. La masse mesurait 18 centimètres de diamètre dans son grand axe; nous avons évité de percuter la masse à cause de la douleur. Les bruits hydro-aériques étaient présents. L'examen au spéculum a montré des muqueuses exo-cervicale et vaginale violacées. Le toucher bi-manuel a retrouvé un col long, postérieur et fermé; l'utérus était augmenté de taille, globuleux et compatible avec une grossesse intra-utérine de dix semaines. L'annexe gauche etait libre et indolore tandis que la douleur empêchait toute palpation bi-manuelle de annexe droite, le cul - de - sac cervico-vaginal droit etait très sensible mais non bombé. Le douglas etait sensible mais non bombé. La culdocentèse était négative. L'examen des membres était normal.

Les hypothèses diagnostiques ont été les suivantes : torsion d'annexe droite torsion d'un léiomyome sous séreux pédiculé, nécrobiose aseptique d'un myome utérin.

Une échographie pelvienne non couplée au doppler a montré un kyste ovarien droit bilobé de 10 centimètres de diamètre sous tension, sans végétations, à parois fines ; aucun épanchement pelvien ni aucun myome n'ont été retrouvés. L'ovaire gauche était d'aspect normal. Une grossesse monofoetale intra-utérine de 8 semaines et 4 jours.

Le diagnostic de travail a été celui d'une torsion de kyste ovarien en grossesse. L'indication de laparotomie urgente a été posée, le bilan pré -opératoire réalisé en urgence était normal (hémogramme, groupages sanguins ABO et Rhésus, bilan d'hémostase). Faute de moyens financiers de la part de la patiente, l'intervention a eu lieu 12 heures plus tard après le début dune supplémentation en progestérone. Les trouvailles étaient: annexe droite (ovaire droit kystique bi-loculé à paroi épaisse de quatorze centimètres de diamètre et à contenu liquidien clair) noire et d'aspect nécrotique tordue à 360° degrés autour du ligament utéro-ovarien, un utérus globuleux augmenté de volume comme pour une grossesse de dix semaines, l'annexe gauche d'aspect macroscopiquement normal, le reste du pelvis sans particularité.

**Figure 1:**
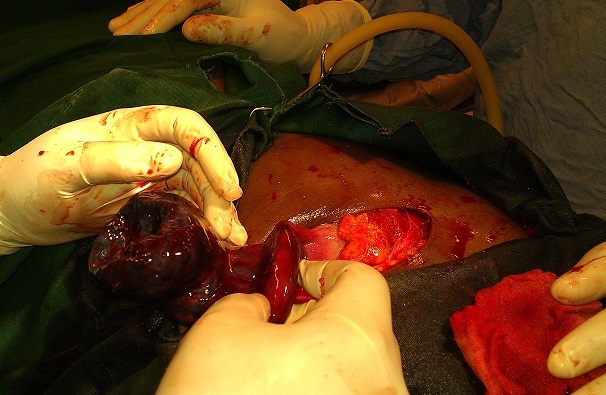
Aspect macroscopique de l'annexe après ablation

**Figure 2:**
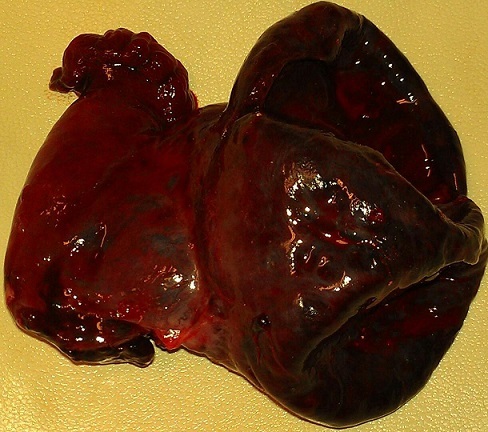
Aspect per-opératoire de l'annexe trente minutes après la détorsion

**Figure 3:**
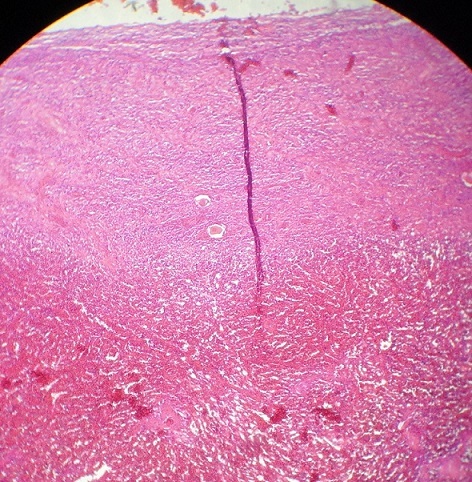
Aspect microscopique après coloration à l'hématéine-éosine

Le geste chirurgical a consisté d'abord en une détorsion de l'annexe, qui n'a montré aucun signe de vitalité 30 minutes plus tard (Figure 1). Nous avons par la suite procédé à une annexectomie gauche (Figure 2). L'évolution a été marquée par la survenue d'un avortement au cinquième jour postopératoire, ceci malgré une tocolyse parentérale à base de caproate d'hydroxyprogestérone. La patiente a quitté l'hôpital au sixième jour post opératoire. Sur le plan anatomopathologique, on a observé à la microscopie un infarcissement hémorragique du tissu ovarien avec foyers de nécrose ischémique associés à des dépôts de fibrine faisant conclure à une apoplexie ovarienne (Figure 3).

## Discussion

La torsion d'annexe est une entité nosologique assez rare qui ne survient au cours de la grossesse que dans 17% à 19,8 % des cas environ [8]. Dans ce cas, soit la masse annexielle (le plus souvent ovarienne) préexiste à la grossesse soit elle apparait et se développe au cours de celle-ci. Dans le premier cas de figure, il s'agit le plus souvent de masse ovarienne de nature organique tandis que dans le second, le caractère fonctionnel est d'autant évident que l'apparition de la masse est favorisée par la stimulation ovarienne dans le cadre de la procréation médicalement assistée et qu'elle disparait spontanément avant la seizième semaine de grossesse [1, 2, 9]. Chez notre gestante, dont la grossesse était spontanée, il s'agissait d'une masse apparue au cours de la grossesse. La torsion dans notre cas s'est produite au cours du premier trimestre en conformité avec les données de la littérature [3, 4].

Le tableau clinique de notre gestante correspondait à la forme-type décrite dans la littérature [10]. Ceci explique, du moins en partie, que le diagnostic clinique correct ait été posé d'emblée alors que la littérature rapporte une exactitude diagnostique à la clinique dans seulement 57,8% des cas [8]. Les principaux diagnostics différentiels devant cette douleur pelvienne intense unilatérale de survenue brutale sans fièvre, sans saignement per vaginal ou intra-péritonéal, sans signes d'occlusion intestinale en présence d'une masse annexielle homolatérale à huit semaines de grossesse sont la torsion d'un léiomyome pédiculé, la colique néphrétique sur lithiase de la jonction urétéro-vésicale ou par compression extrinsèque du bas uretère [10]. Ces deux diagnostics ont été éliminés par l'échographie pelvienne réalisée aux urgences qui a permis de préciser la nature kystique de la masse et d'exclure une dilatation de la voie excrétrice.

La prise en charge chirurgicale des masses ovariennes au cours de la grossesse ne se conçoit que dans deux situations: la survenue de complications aiguës telles que la torsion, la rupture ou l'hémorragie intra-kystique, et la présence d'arguments de malignité ou simplement la persistance d'un kyste d'allure bénigne au-delà de la quatorzième semaine d'aménorrhée. La voie d'abord initiale avant la seizième semaine de grossesse est coelioscopique que la chirurgie soit urgente ou programmée. Elle permet assez souvent de réaliser le geste opératoire en laissant possible la conversion en laparotomie. Le geste thérapeutique dépend de l'aspect de la masse; une approche conservatrice consistant en une détorsion associée ou non à une ovariopexie est logique devant une masse d'aspect viable. L'ovariectomie ou l'annexectomie est indiquée devant une masse d'allure nécrotique ; pour certains auteurs cependant, elle n'est indiquée que devant des arguments macroscopiques de malignité. Pour ces derniers, la grande capacité de récupération fonctionnelle du tissu ovarien justifie d'être conservateur même devant une annexe de vitalité douteuse [2, 7, 11–16]. Dans le cas que nous présentons, la chirurgie n'a été réalisée que vingt-quatre heures après le début des symptômes à cause des faiblesses institutionnelles qui caractérisent notre contexte pauvre en ressources. Faute de plateau technique approprié et de compétence en coeliochirurgie nous avons réalisé une laparotomie ; pourtant la chirurgie laparoscopique a fait la preuve de son efficacité dans cette indication [7, 12, 13, 15]. Devant l'aspect nécrotique de l'annexe tordue et compte tenu des difficultés contextuelles nous avons pratiqué une annexectomie; la malade et ses proches avaient au préalable été informés de cette possibilité et du risque de fausse couche encouru. Pour prévenir cette complication, un traitement parentéral à base de caproate de progestérone a été initiée avant la chirurgie ; quoique bien conduite elle n'a pas permis d'éviter la survenue d'une fausse couche au cinquième jour post opératoire. Opérée plus tôt, une détorsion simple aurait peut être permis de conserver ce corps jaune gravidique et de poursuivre la grossesse. Comme la plupart des kystes ovariens rencontrés au premier trimestre de la grossesse, le notre était fonctionnel comme l'a confirmé l'analyse histologique qui a mis en évidence une apoplexie ovarienne (Figure 3). L'activité sécrétoire intense du corps jaune entraine une augmentation de son volume et de sa vascularisation en qualité et en quantité ; ceci pourrait expliquer l'apoplexie observée dans notre cas [11]. La survenue de la fausse couche malgré une tocolyse parentérale bien conduite rappelle le rôle primordial du corps jaune au premier trimestre de la grossesse.

## Conclusion

L'issue défavorable de cette grossesse après l'ablation chirurgicale de son corps jaune tordu souligne l'importance du traitement conservateur des kystes lutéaux lorsque ceux-ci se compliquent au cours du premier trimestre. La précocité de la chirurgie après le début des symptômes augmente les chances préservation du corps jaune mais n'est pas toujours possible dans notre contexte aux ressources très limitées.

## References

[1] Schwartz N, Timor-Tritsch IE, Wang E (2009). Adnexal masses in pregnancy. Clin Obstet Gynecol..

[2] Schmeler KM, Mayo-Smith WW, Peipert JF, Weitzen S, Manuel MD, Gordinier ME (2005). Adnexal masses in pregnancy: surgery compared with observation. Obstet Gynecol..

[3] Chang SD, Yen CF, Lo LM, Lee CL, Liang CC (2011). Surgical intervention for maternal ovarian torsion in pregnancy. Taiwan J Obstet Gynecol..

[4] Erdemoglu M, Kuyumcuoglu U, Kale A (2010). Pregnancy and adnexal torsion: analysis of 20 cases. Clin Exp Obstet Gynecol..

[5] Rackow BW, Patrizio P (2007). Successful pregnancy complicated by early and late adnexal torsion after in vitro fertilization. Fertil Steril..

[6] Oelsner G, Shashar D (2006). Adnexal torsion. Clin Obstet Gynecol..

[7] Toure B, Dao B, Sano D, Akotionga M, Lankoande J, Kone B (1997). Adnexal torsion during pregnancy: Diagnostic and therapeutic problems in Burkina Faso. Rev Med Brux..

[8] Bouguizane S, Bibi H, Farhat Y (2003). Adnexal torsion: a report of 135 cases. J Gynecol Obstet Biol Reprod (Paris).

[9] Hasiakos D, Papakonstantinou K, Kontoravdis A, Gogas L, Aravantinos L, Vitoratos N (2008). Adnexal torsion during pregnancy: report of four cases and review of the literature. J Obstet Gynaecol Res..

[10] Marret H, Laffo M, De Calan L, Bourlier LP, Lansac J (2000). Urgences chirurgicales au cours de la grossesse. Encycl Méd Chir. Gynécologie/Obstétrique.

[11] Chauveaud-Lambling A, Picone O, Fernandez H (2006). Tumeurs de l'ovaire et grossesse. Encycl Méd Chir, Gynécologie/Obstétrique.

[12] Busine A, Murillo D (1994). Conservative laparoscopic treatment of adnexal torsion during pregnancy. J Gynecol Obstet Biol Reprod (Paris).

[13] Morice P, Louis-Sylvestre C, Chapron C, Dubuisson JB (1997). Laparoscopy for adnexal torsion in pregnant women. J Reprod Med..

[14] Bider D, Mashiach S, Dulitzky M, Kokia E, Lipitz S, Ben-Rafael Z (1991). Surg Gynecol Obstet. Clinical, surgical and pathologic findings of adnexal torsion in pregnant and nonpregnant women..

[15] Moore RD, Smith WG (1999). Laparoscopic management of adnexal masses in pregnant women. J Reprod Med..

[16] Stepp KJ, Tulikangas PK, Goldberg JM, Attaran M, Falcone T (2003). Laparoscopy for adnexal masses in the second trimester of pregnancy. J Am Assoc Gynecol Laparosc..

